# Angry facial expressions bias towards aversive actions

**DOI:** 10.1371/journal.pone.0256912

**Published:** 2021-09-01

**Authors:** Leon O. H. Kroczek, Angelika Lingnau, Valentin Schwind, Christian Wolff, Andreas Mühlberger

**Affiliations:** 1 Department of Psychology, Clinical Psychology and Psychotherapy, University of Regensburg, Regensburg, Germany; 2 Department of Psychology, Cognitive Neuroscience, University of Regensburg, Regensburg, Germany; 3 Human Computer Interaction, University of Applied Sciences in Frankfurt a. M, Frankfurt a. M., Germany; 4 Department of Media Informatics, University of Regensburg, Regensburg, Germany; University Hospitals Tubingen: Universitatsklinikum Tubingen, GERMANY

## Abstract

Social interaction requires fast and efficient processing of another person’s intentions. In face-to-face interactions, aversive or appetitive actions typically co-occur with emotional expressions, allowing an observer to anticipate action intentions. In the present study, we investigated the influence of facial emotions on the processing of action intentions. Thirty-two participants were presented with video clips showing virtual agents displaying a facial emotion (angry vs. happy) while performing an action (punch vs. fist-bump) directed towards the observer. During each trial, video clips stopped at varying durations of the unfolding action, and participants had to recognize the presented action. Naturally, participants’ recognition accuracy improved with increasing duration of the unfolding actions. Interestingly, while facial emotions did not influence accuracy, there was a significant influence on participants’ action judgements. Participants were more likely to judge a presented action as a punch when agents showed an angry compared to a happy facial emotion. This effect was more pronounced in short video clips, showing only the beginning of an unfolding action, than in long video clips, showing near-complete actions. These results suggest that facial emotions influence anticipatory processing of action intentions allowing for fast and adaptive responses in social interactions.

## Introduction

Social interaction requires the understanding of action intentions. In face-to-face situations, it is crucial to capture another person’s intentions as fast and accurately as possible in order to generate an adaptive response. Recognizing action intentions is thus necessary to ensure that interactive behavior is coordinated in space and time [1]. Typically, this task is mastered effortlessly in our everyday lives, for instance, every time when we have to reciprocate a greeting of another person or when we have to reach for an object that is offered to us [2]. According to motor simulation theories, observation of an action activates the corresponding motor program in the observer’s motor system and thus enables action recognition (for an overview see [3]). This view, however, has been challenged by studies that observed no direct evidence for direct motor involvement in action observation and rather suggest an influence of higher level conceptual representations in action recognition [4–9]. Recent models have highlighted the role of hierarchical predictive processes in action understanding [10–13]. According to such models, people use internal models, prior experience as well as context information in order to predict upcoming actions. Predictive processing therefore allows anticipating of actions and reacting to them in a fast and efficient manner. Importantly, both predictive processing and conceptual accounts highlight the role of multimodal information in action intention processing.

In face-to-face settings, multiple sources of information are available that can be used to anticipate an upcoming action. Previously, it has been demonstrated that observers process the preshaping of the hand [14], gaze-direction [15], as well as kinematic information (e.g. wrist height or wrist trajectory [16]) in order to infer action intentions for upcoming movements. Importantly, there is also evidence that observers integrate multimodal sources of information during action observation. For instance, Ambrosini et al. [17] reported that perceived gaze direction of the agent of an action influenced the processing of the intention of the action, but only as long as no reliable information on hand-shape was available. This finding suggests that the influence of observed gaze direction may be restricted to early phases of an unfolding action whereas during later phases, observers seem to rely on additional sources of information. It has to be noted, however, that previous studies mostly investigated object-related actions, while social interactions often entail person-related actions (e.g. handshake or fist bump). Given that person-related actions are typically accompanied by social cues, such as (direct) eye gaze or facial expressions [18], it seems plausible that social cues have an even stronger influence on person-related than object-related actions. Furthermore, previous studies found evidence that action recognition is influenced by social information such as the identity [19] or the gender of the actor [20–22].

Crucially, real-life social interactions provide a range of informative cues besides eye-gaze, such as body posture and facial emotions [23,24]. Emotional expressions of the face are highly salient and communicative cues in social interaction [18,25]. Importantly, facial emotions communicate threat or affiliative tendencies in social encounters [26] and therefore may be used to infer action intentions and predict upcoming behavior. For example, punches might be more likely when a person is showing an angry facial emotion, whereas handshakes should be more likely when a person is showing a happy facial emotion. Previous studies investigating the link between emotion and action have found evidence for an emotional modulation of motor responses. For instance, this modulation could be demonstrated in terms of increased motor excitability in the presence of emotional compared to neutral stimuli [27], and in terms of altered neural activation of motor-related brain areas by emotional stimuli [28,29]. Furthermore, there is evidence for increased functional connectivity between the amygdalae and premotor areas during observation of emotional compared to neutral faces [30]. A recent study demonstrated that the effective connectivity between the amygdala and insula drives the processing of emotional body language [31]. The insula has also been related to top-down processing during the perception of biological motion [32] and may thus drive the emotional influence on action perception. These findings are further in line with the view that processing of social threat cues (e.g. in the face or body) results in preparatory activation that allows for adaptive action [33]. Only scarce evidence, however, is available with respect to the influence of facial emotions on the understanding of action intentions. Shih and Lin [34] reported a positive correlation in performance between facial emotion recognition and action anticipation in a group of taekwondo athletes, thereby providing indirect evidence for a link between facial emotions and action recognition. However, because the study only investigated sport-related actions it is not clear whether this effect also relates to everyday social interactions.

The goal of the present study was to investigate whether facial emotions influence the processing of action intentions, and if so, whether this influence is modulated by the amount of information that is available. To this aim, we presented video clips of two virtual agents (one male, one female) showing facial emotions (angry vs. happy) and performing actions (punch vs. fist-bump) directed towards the observer. Facial emotions and actions were chosen to be either appetitive (happy facial emotion/fist bump action) or aversive (angry facial emotion/punch action). Importantly, video clips differed in their duration, stopping at various stages of completeness of the unfolding action. Consequently, actions were harder to recognize with decreasing length of the video clip. After every video presentation, participants answered a two-alternative forced-choice task where they had to indicate which action they had recognized in the video.

We hypothesized to find an effect of facial emotions on action intention processing. In principle, such an effect could work in two different ways. On the one hand, facial emotions might affect the general accuracy in recognizing actions, i.e. correctly recognizing punches as punches and fist bumps as fist bumps. On the other hand, facial emotions might bias participants’ response towards congruent choices (i.e., judging an action as ‘fist bump’ if the facial emotion was happy, and judging an action as ‘punch’ if the facial emotion was angry), regardless of the actual presented action. We tested the first claim by investigating participants’ ability to discriminate between the two actions, and the latter by investigating proportions of ‘punch’ judgements in fist bump stimuli and punch stimuli respectively. We evaluated both measures as a function of facial emotion and video clip duration. While we expected that recognition accuracy should increase with video duration, we hypothesized that facial emotions would affect action judgements regardless of the actual presented action. In particular, an angry compared to a happy facial emotion should increase the likelihood to judge an action as a punch, for both punch and bump stimuli. Likewise, a happy compared to an angry facial emotion should increase the likelihood to judge an action for a fist bump, for both punch and bump stimuli. This effect should be strongest when actions are hard to recognize (i.e. short video clips) and less prominent when actions are easy to recognize (i.e. long video clips).

## Materials and methods

### Participants

Thirty-two healthy students who did not report any mental or neurological diseases participated in the present study (M_age_ = 22.65, SD_age_ = 3.72, range = 19–40 years, 23 female). The sample size allowed to detect medium effect sizes of δ ~ 0.5 with a power of 1-β = 0.80 (determined for paired t-tests and alpha = 0.05). Participants gave written informed consent and received credit points as compensation. Experimental procedures were in line with the Declaration of Helsinki, and the study was approved by the ethics board of the University of Regensburg. The study was conducted according to the approved guidelines.

### Stimulus material

**Fig 1:**
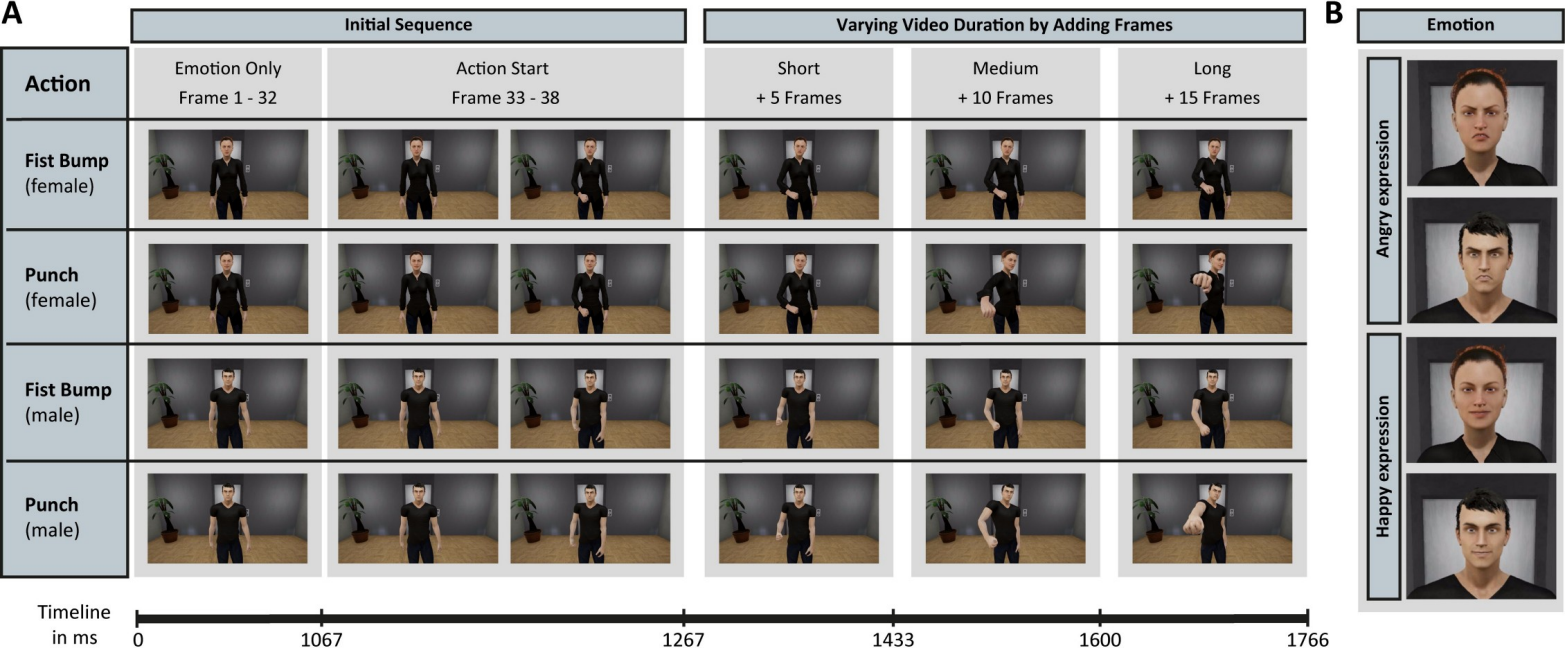
Schematic illustration of the video stimuli. (A) Each row depicts the timeline of an unfolding action (fist bump or punch), separately for the two agents (female and male). Two exemplary combinations of facial emotions and actions are shown per agent. The first three columns show the initial sequence that was presented in all video stimuli (frames 1–38). Agents displayed the facial emotion starting from the first frame (the facial emotion remained constant throughout the video clip). The initial sequence also included the onset of the action, from frame 33 to frame 38. From frame 39 onwards, single frames were added to the video clips in order to vary the degree of completeness to which an unfolding action was presented. Video duration was grouped according to the number of added frames, resulting in ‘short’ (1–5 frames added), ‘medium’ (6–10 frames added), and ‘long’ (11–15 frames added) video durations. (B) Illustration of the angry and happy facial emotions of both agents. Reprinted from https://www.daz3d.com/under a CC BY license, with permission from Daz Productions Inc, original copyright 2021.

Stimulus material consisted of short video clips which depicted one of two virtual agents in a virtual room displaying a facial emotion (angry vs. happy) and performing an action (punch vs. fist bump). Virtual agents were shown from a frontal position and the actions were directed towards the viewer. Importantly, we varied the duration of these video clips videos by stopping them at different end-frames while the action was unfolding. More specifically, videos differed with respect to the number of frames that were presented after a fixed initial period. This initial period contained the facial emotion and the onset of the action (see Fig 1).

For stimulus creation, a female and male actor performed two distinct actions, a fist bump and a punch, while their body movements were recorded via a OptiTrack motion capturing system (12 cameras: 8 PRIME 13 and 4 PRIME 13W) tracking a total of 55 optical markers (39 passive body, 16 active finger marker) on a full-body motion tracking suit. Movements and skeleton animation were recorded using Motive software (v 2.2) and then preprocessed in Autodesk 3ds Max (v 2019) by reducing the number of keyframes (from 240 to 30 fps) for automatic noise reduction. Finally, animations of both actions were aligned at a reference frame and the ten initial frames of the actions were averaged, resulting in ten similar, albeit not identical, frames for both animations. This procedure was performed separately for the male and the female animations. Finally, we created three different exemplars per action by inducing slight variations with respect to the end position (vertical and horizontal offset) of each action. All animations were exported into the Unreal Engine (v 4.22, Epic Games).

In a next step, two virtual agents (one male, one female) were created using Daz3D (Daz3D Inc.), based on the standard Genesis 8 models. They received black clothes and standard geometry-based hair. The agents were imported into the Unreal Game Engine 4 and placed into a virtual room in front of an elevator door. Morph targets using Daz3D and the processed mocap animation sequences using Unreal action blueprints were created in which the agents first showed facial emotions (angry or happy) and then performed one of the animated actions (fist bump or punch). Facial emotions always remained constant while actions were performed.

These animation sequences were used to export video stimuli with 30 fps. Each video started with an initial sequence where the facial emotional expression of the agent was presented for 32 frames (i.e. 1067.7 ms; see Fig 1A, left column) followed by the onset of the action for 6 frames (i.e. 200 ms; see Fig 1A, 2^nd^ and 3^rd^ column). Thus, the initial sequence had a length of 1267.7 ms. From that point on, i.e. from 1266.7 ms onwards, we varied the number of frames (1–15) that were presented to the participant, resulting in 15 different video clip lengths per condition.

Importantly, fist bump and punch actions started from identical positions and body configurations (see Fig 1A, 2^nd^ column), and the trajectories of both actions diverged as more frames were added to the video clips which showed the unfolding actions. Naturally, both actions were similar at early stages of the unfolding actions and more distinct at late stages of the unfolding actions.

In total, there were 2 (agent gender: female/male) by 2 (emotion: angry/happy) by 2 (action: punch/fist bump) by 3 (exemplars) by 15 (video clip duration) = 360 video stimuli. Video length differed according to the number of added frames between 1300 ms (1 frame added) and 1766 ms (15 frames added).

### Procedure

Participants were seated in front of a 21.5-inch LCD-screen (HP E221c, 1920x1080 resolution, 60 Hz) with a distance of 50 cm. Stimulus presentation was controlled using PsychToolbox [35] in Matlab (MathWorks). Video clips were centered on the screen with a size of 1520x855 pixels (visual angle: 41.30° x 23.96°). In total, 360 trials were presented in a pseudo-randomized order with no more than three repetitions of agent gender, emotion or action. Each trial started with the presentation of a fixation cross for 1000 ms, followed by the video clip (1300–1766 ms). After the video clip, a two-alternative forced choice task was presented where participants were asked to indicate which action they had recognized in the previous video via button press on the keyboard with the left or right index finger. Response options (“fist bump”/”punch”) were presented on the left and right side of the screen, respectively. Within participants, these response options were always presented on the same side across trials. Across participants, side was counterbalanced. After a response was given, the next trial started. Total experimental duration was about 20 minutes.

### Data processing and statistical analysis

Statistical analysis was performed using R (v 4.0.2, [36]). In order to investigate the effect of video duration, we grouped trials according to the number of added frames in the video clips, resulting in short (1–5 frames added), medium (6–10 frames added) and long (11–15 frames added) conditions. These conditions were entered as levels of the factor *video duration* in subsequent statistical analyses (visualizations of aggregated data are presented in **S1 and S2 Figs**). Furthermore, we grouped trials across agent gender and across versions of the same action, resulting in 2 (*emotion)* by 2 (*action)* by 3 (*video duration)* experimental conditions. Each condition contained data from 30 trials per participant.

In a first step, we analyzed participants’ recognition accuracy for both punch and fist bump actions. Therefore, we calculated d-prime as a discriminability index [37]. The proportion of correctly recognized punch actions (response = punch and stimulus = punch; hit rate) and the proportion of falsely recognized punch actions (response = punch and stimulus = fist bump; false alarm rate) was determined and a loglinear adjustment for extreme values was applied [38]. D-prime was calculated by subtracting the z transform of the false alarm rate from the z transform of the hit rate: d’ = z(hit rate)–z(false alarm rate). Individual d-prime values were entered into a repeated-measures ANOVA using the within-subject factors *emotion* (levels: angry, happy) and *video duration* (levels: short, medium, long). D-prime values in all conditions were approximately normally distributed, as assessed by the Shapiro-Wilk-Test (all W > .95, all p > .05).

The next analysis focused on a potential bias in action recognition with respect to facial emotion and the actual performed action for different video durations. For that reason, the proportion of trials where participants judged an action as a ‘punch’ (regardless of whether correct or incorrect) was analyzed using a repeated-measures ANOVA with the within-subject factors *emotion*, *action*, and *video duration*. Proportion of punch responses in all conditions were approximately normally distributed, as assessed by the Shapiro-Wilk-Test (all W > .95, all p > .05). For all analyses, assumption of sphericity was tested using Mauchly’s test of sphericity and sphericity violations were corrected according to Greenhouse-Geisser [39], and in these cases epsilon values are reported as well. Post-hoc t-tests were conducted to follow-up on significant effects and corrected for multiple comparisons after Holm [40].

## Results

### Action discriminability

**Fig 2:**
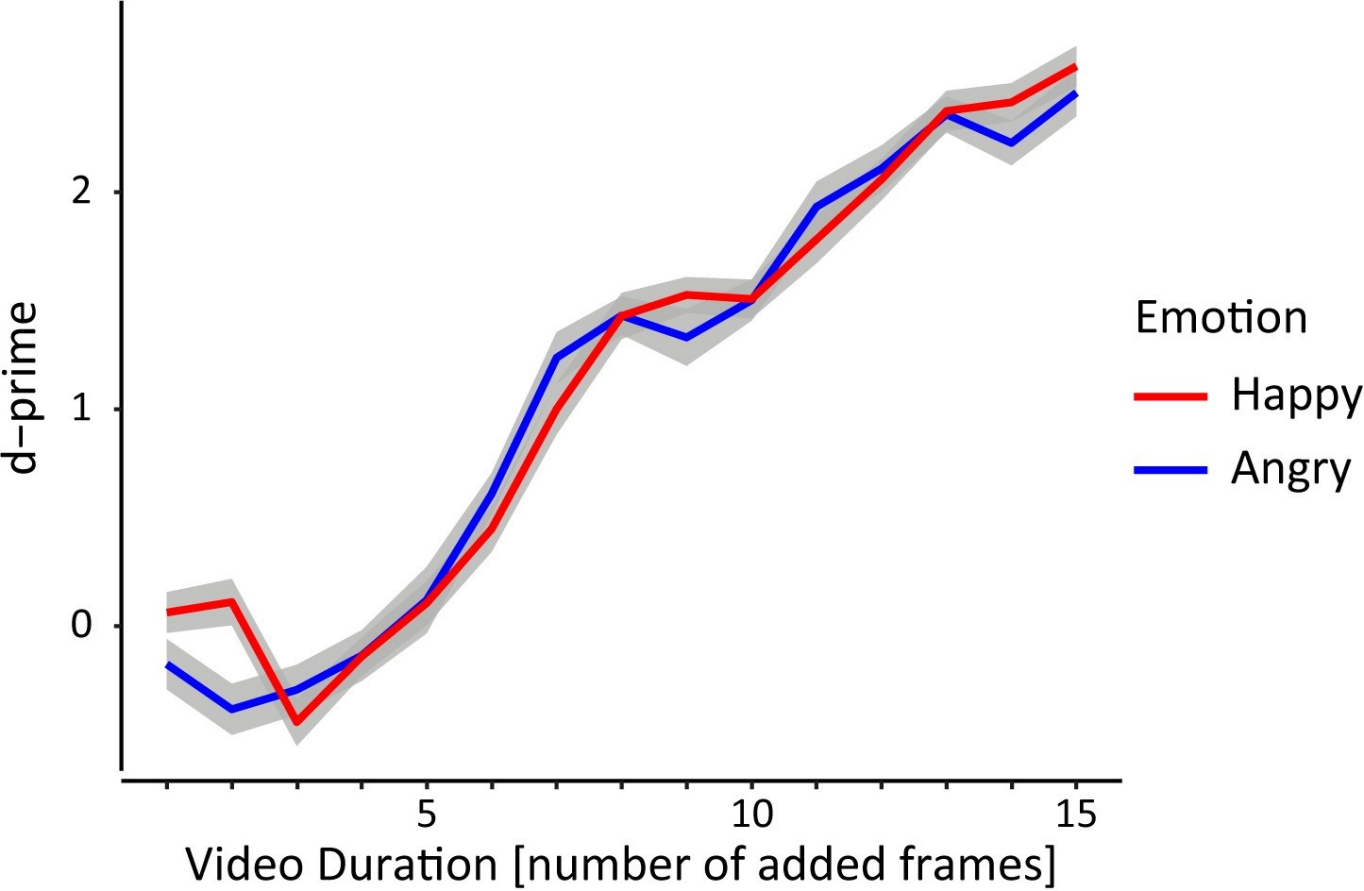
D-prime as a function of and video duration and facial emotion. D-prime values as a function of video duration in number of added frames and facial emotion of the agents (angry, happy). Shaded areas depict the standard error of the mean.

Individual d-prime values were analyzed using a repeated-measures ANOVA with factors *emotion* and *video duration* (see Fig 2). There was a significant main effect of *video duration*, *F*(2,62) = 581.36, *p* < .001, *η*_*p*_^*2*^ = 0.95, but no main effect of *emotion* F(1, 31) = 1.63, p = .211 and no interaction of *emotion* and *video duration*, F(2, 62) = 0.64, p_GG_ = .500 (ε = 0.81). As expected, participants were significantly better to discriminate between actions in long compared to medium duration video clips, t(31) = 16.97, p < .001, d = 3.00, as well as in medium compared to short duration video clips, t(31) = 20.84, p < .001, d = 3.68.

According to signal detection theory a d-prime of zero indicates that participants are unable to discriminate between two stimuli [37]. Therefore, a further analysis was conducted to evaluate whether d-prime values were significantly different from zero for a given video duration. D-prime values were significantly greater than zero for actions in the medium and long video duration condition, t(31) = 19.70, p < .001, d = 3.48, t(31) = 29.59, p < .001, d = 5.23, respectively. Interestingly, however, d-prime was significantly smaller than zero when actions were presented with short video durations, t(31) = -3.00, p = .005, d = -0.53, indicating that participants were mistaking one action for the other.

In summary, recognition accuracy, i.e. participants’ ability to discriminate between punch and fist bump actions, increased depending on the degree to which an unfolding action was revealed in the video clips. Participants were unsuccessful in correctly discriminating between both actions when only five or less frames of an unfolding action were added in the video clips. Furthermore, facial emotions of the virtual agents did not influence discriminability between actions.

### Emotion bias

**Fig 3:**
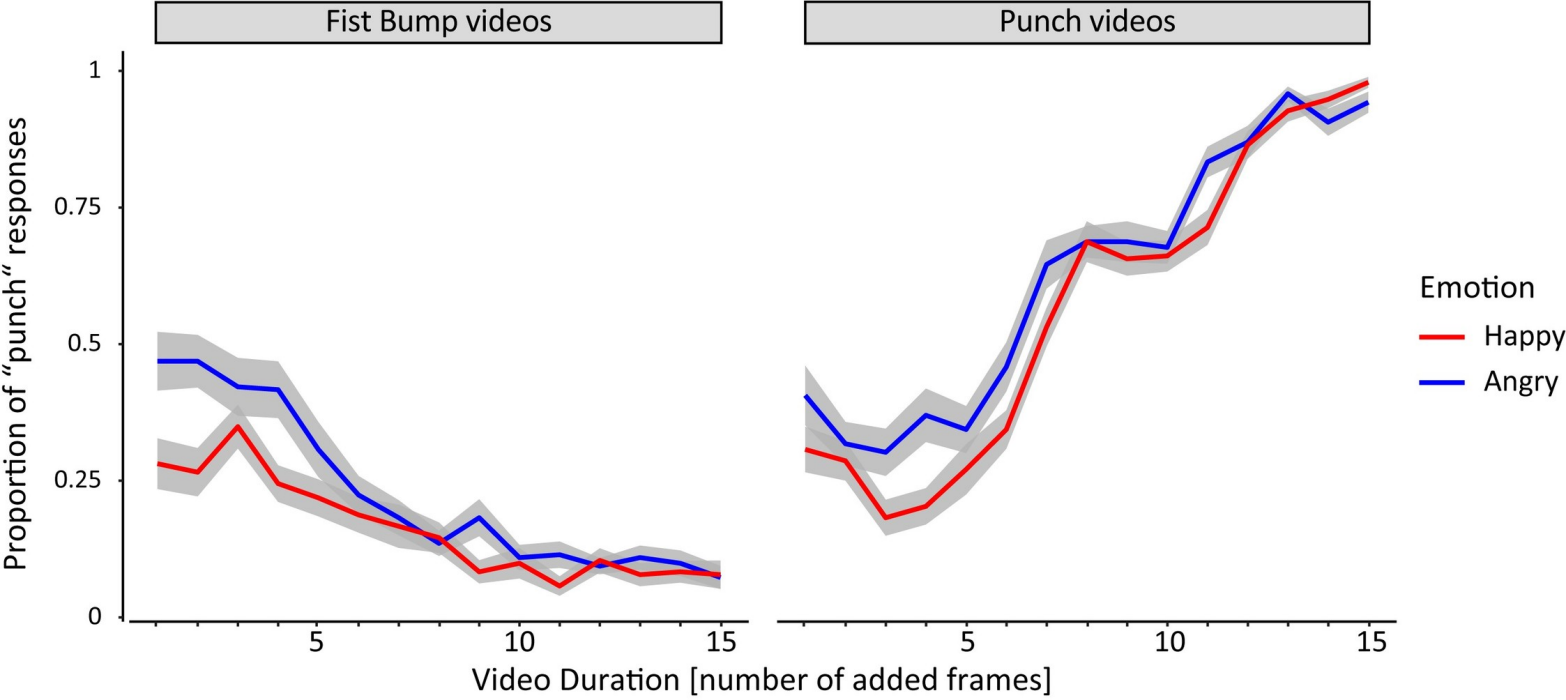
Action judgments as a function of action, facial emotion and video duration. Proportion of punch responses for different facial emotions (angry, happy), separately for the two different actions (fist bump, punch), as a function of video duration in number of added frames. Shaded areas depict the standard error of the mean.

In order to test whether facial emotions affect participants’ action judgements, we analyzed the proportion of trials where participants judged an action as a ‘punch’ (correctly or incorrectly) depending on the facial *emotion*, the actual performed *action*, and the *video duration* (Fig 3). Participants judged both fist bump and punch actions more frequently as punches when agents displayed an angry facial emotion compared to when agents displayed a happy facial emotion. This effect was strongest for short video durations.

A repeated-measures ANOVA revealed a three-way interaction of *emotion*, *action*, and *video duration*, F(2,62) = 3.25, p = .046, *η*_*p*_^*2*^ = 0.09, as well as significant two-way interactions of *emotion* by *video duration*, F(2, 62) = 11.08, p < .001, *η*_*p*_^*2*^ = .26, and of *action* by *video duration*, F(2, 62) = 781.80, p < .001, *η*_*p*_^*2*^ = .96. There were also significant main effects of *emotion*, F(1, 31) = 23.74, p < .001, *η*_*p*_^*2*^ = .43, *action*, F(1, 31) = 1151.27, p < .001, *η*_*p*_^*2*^ = .97, and *video duration*, F(2, 62) = 32.65, p_GG_ < .001, *η*_*p*_^*2*^ = .51 (ε = 0.63). In order to resolve the interaction effects involving *video duration*, step down analyses were conducted for short, medium, and long video clip duration separately.

For short video durations there was a significant interaction of *emotion* by *action*, *F*(1,31) = 6.68, p = .015, *η*_*p*_^*2*^ = 0.18, as well as a main effect of *emotion*, *F*(1,31) = 22.90, *p* < .001, *η*_*p*_^*2*^ = 0.42, and a main effect of *action*, *F*(1,31) = 10.07, *p* = .003, *η*_*p*_^*2*^ = 0.26. Post-hoc t-tests revealed that the proportion of actions judged as punch actions was greater when agents displayed an angry facial emotion compared to when agents displayed a happy facial emotion. This pattern was observed, regardless of whether the video stimulus actually showed a punch, t(31) = 3.72, p = .002, d = 0.66, or a fist bump, t(31) = 5.27, p < .001, d = 0.93. Interestingly, when agents displayed an angry facial emotion, participants reported punch actions more frequently for video clips showing fist bump actions compared to video clips showing punch actions, t(31) = 3.93, p = .001, d = 0.69. There was no significant difference between punch and fist bump video clips when agents were displaying a happy facial emotion, t(31) = 1.40, p = .171, d = 0.25.

For medium video clip durations, a significant main effect of *emotion* was observed with a larger proportion of punch judgements for angry compared to happy emotions, *F*(1,31) = 6.51, *p* = .016, *η*_*p*_^*2*^ = 0.17. Furthermore, there was a main effect of *action*, F(1, 31) = 504.93, p < .001, *η*_*p*_^*2*^ = .94, as participants reported punch actions more frequently for punch video clips compared to fist bump video clips. There was no interaction of *emotion* by *action*, F(1, 31) = 1.28, p = .267, η_p_^2^ = .04.

For long video clip durations, we observed a significant main effect of *action*, F(1, 31) = 2199.15, p < .001, *η*_*p*_^*2*^ = .99, with punch actions being reported more frequently for punch video clips compared to fist bump video clips. There was only a tendency for the effect of *emotion*, F(1, 31) = 3.88, p = .058, *η*_*p*_^*2*^ = .11. According to this tendency, punch actions were reported more frequently when agents showed an angry facial emotion compared to a happy facial emotion. Again, there was no interaction of *emotion* by *action*, F(1, 31) = 0.02, p = .896, *η*_*p*_^*2*^ < .01.

Finally, we compared the effect of emotion, i.e. the difference in punch responses between the angry emotion condition and the happy emotion condition, between different video durations. The emotion effect was significantly greater for short video durations compared to medium video durations, t(31) = 3.08, p = .008, d = 0.54, and long video durations, t(31) = 4.27, p < .001, d = 0.75. There was no significant difference between the emotion effect at medium and long video durations, t(31) = 1.39, p = .175, d = 0.25.

In summary, facial emotions influenced participants’ judgements regarding recognized actions. Angry facial emotions increased the proportion of punch judgements compared to happy facial emotions. This effect was most prominent at short and medium video durations and was observed regardless of whether the actual presented action in the video stimulus was a punch or a fist bump. Interestingly, for short video durations, punch judgements were most frequent when an angry agent produced a fist bump action.

## Discussion

The present findings show that facial emotions bias the processing of action intentions. Angry compared to happy facial emotions increased participants’ likelihood to judge a presented action as an aversive punch action (emotional bias effect). Interestingly, while facial emotions biased participants’ choices, they did not affect recognition accuracy across actions. Furthermore, the emotional bias effect was most prominent at short video durations, when participants were not able to discriminate correctly between actions. Importantly, however, the influence of emotion was still present at medium video durations, when participants’ recognition accuracy improved for both actions. This indicates that facial emotions influence the processing of action intentions, even when actions can be recognized by hand trajectory or preshaping. These results highlight the role of facial emotions in action processing.

Facial emotions provide contextual information in social encounters, which allow an observer to infer action intentions and to anticipate actions. This is in line with previous findings related to contextual effects on the recognition of action intentions, for example via eye gaze [14,17], and supports predictive processing accounts of action recognition where multimodal cues are used to predict upcoming actions [10]. As an alternative interpretation it is possible that the perception of a facial emotion activates higher-level conceptual representations of social actions [41] that are compatible with these emotions (e.g. ‘fighting’ when seeing an angry facial emotion). As these conceptual representations are linked to specific actions, e.g. a punch, they could allow for a co-activation of action by emotional cues. Because emotional cues typically indicate highly relevant, e.g. aversive or appetitive behavior, they might be even weighted more strongly than non-emotional cues in generating predictions or activating conceptual representations. Importantly, however, the link between emotion and action might be influenced by additional factors, such as person-related or situation-related information [19,42]. For instance, Hudson et al. [42] could show that an actor’s statements about the action goal modify action perception. In sum, the present data suggest that observers are sensitive to social cues that allow anticipating an upcoming action and that they use this information in order to extract action intentions. This mechanism allows the fast and efficient interactive behavior that is required for adaptive responses in social encounters.

Crucially, while the emotional bias effect was most prominent at short video durations, facial emotions still influenced participants’ responses at medium video durations (for long video durations there was only a tendency). This finding suggests that facial emotions are highly salient cues that impact action intention processing even when actions can be easily recognized on the basis of other information like hand trajectory or preshaping. This is in contrast to findings by Ambrosini et al. [17] who found that the influence of multimodal cues, such as an actor’s gaze behavior, was restricted to early stages of an unfolding action when no informative motor cues were available. Importantly, however, Ambrosini et al. investigated object-related actions (i.e. grasping) while the present study focused on person-related actions (e.g. punches and fist bumps). With respect to the latter, processing of socio-emotional information might be highly relevant even in the presence of additional information provided by hand trajectory or preshaping.

An unexpected finding of the present study was that, for short video durations, participants reported more punch actions in videos showing fist bump actions compared to videos showing punch actions when agents displayed an angry facial emotion. There was no difference between punch and fist bump conditions when agents showed a happy facial emotion. Thus, at short video durations, the emotional bias effect was greater for fist bump videos compared to punch videos. This effect, however, should be treated with caution, as fist bump actions and punch actions were not identical at these short video durations. Therefore, the observed difference might be based on subtle variations in the video stimuli which are relevant in combination with the displayed emotion. Importantly, this finding did not obscure the emotional bias effect, as angry expressions increased punch judgments compared to happy expressions for both fist bump and punch actions.

It should also be noted that overall participants reported more fist bump actions than punch actions (especially at short video durations). This was possibly due to the fact that both actions were initiated from a low starting position, with the agent’s hand in a relaxed neutral position next to the legs (cf. Fig 1A, 2^nd^ column), while the end position of the action was lower for the first bump in comparison to the punch action (cf. Fig 1A, 6^th^ column). Thus, the starting position was spatially closer to the end position of the first bump than to the end position of the punch action. This might explain the general bias to report a fist bump action when the unfolding action was stopped after only a few frames, i.e. when the agent’s hand was still at a low position. In line with this interpretation, previous studies have reported a strong influence of hand preshape and arm movement on processing of action intentions [17]. An alternative explanation for the overall bias towards fist bump actions could be that participants used world-knowledge and social norms in order to generate general expectations for action intentions. In real life, appetitive actions are much more frequent and accepted than aversive actions, i.e. greetings are much more common than punches. Importantly, however, participants’ general tendency to judge an action to be a fist bump did not influence the observed effect of facial emotions.

To our knowledge, the present study is the first to show effects of facial emotion on the processing of action intentions. However, there are limitations that need to be discussed. First, the range of actions was limited to fist bump and punch and the range of facial emotions was limited to happy and angry expressions. This does not reflect the behavioral variety in real social interactions and might have increased the saliency of the presented actions. Note, however, that there was variation with respect to the agents’ actions as agents were animated from real movement recordings stemming from two different persons. While future studies should present a wider range of actions, we believe it is unlikely that this would affect our main finding, i.e. the emotional bias for action recognition. The facial emotions and the actual actions were presented in a crossed design, i.e. a happy emotion was presented with a fist bump action for the same number of trials as it was presented with a punch action. Therefore, the facial emotion was not informative with respect to action. Furthermore, the experimental task only required participants to report the action and no feedback was given regarding whether the response was correct. Importantly, despite being uninformative and task-irrelevant, facial emotions still influenced participants’ responses.

Another limitation is the use of virtual agents, which might have been less realistic than real actors. Note, however, that the presented actions were recorded from real persons via motion tracking. The use of a virtual scenario allowed for high experimental control in combining these actions with facial emotions. This would have been hard to achieve with real actors. Furthermore, previous studies found similar neural responses related to the processing of emotions in natural and artificial faces [43,44] and were successful in replicating behavioral effects of social interaction in Virtual Reality [45].

Finally, the present study did not investigate gender effects. Previous studies found gender effects both related to the gender of the observer [20,21] as well as the gender of the actor [21,22]. Such effects might also affect the influence of facial emotions on the processing of action intentions. In the present study, however, the sample size regarding male was too small (n = 9 male participants) to reliably estimate gender effects with respect to the observer. Future studies with greater and gender-balanced samples should address this question. With respect to the actor, we did not include gender as a factor in our analysis because the presented actions were generated from the individual movements of a male and female actor and thus gender was confounded with the kinematic properties of the individual actions (see [46]). In order to study effects of actor gender, future studies might use stimuli where action kinematics are kept constant between male and female agents. Alternatively, including several male and female agents might allow examining variability between individual actions within the same gender.

Taken together, the present study investigates the influence of facial emotional expressions on action intention processing. We observed an emotional bias effect, where an angry compared to a happy facial emotion increased the likelihood to judge an action as aversive. This effect was most prominent when action recognition accuracy was low, but was retained even when actions could be easily recognized on the basis of arm movements or preshaping of the hand. These findings highlight multimodal information processing as an adaptive mechanism for inference of action intentions during social interactions.

## Supporting information

S1 Fig Action discriminability: Data averaged for video duration conditions. Averaged d-prime values as a function of video durations (short, medium, long) and facial emotions of the agents (angry, happy). Video duration conditions were aggregated on basis of the number of added frames. Short duration relates to 1–5 added frames, medium duration to 6–10 added frames and long duration to 11–15 added frames. Error bars depict the standard error of the mean.
(TIF)

S2 Fig Emotion bias: Data averaged for video duration conditions. Proportion of punch responses as a function of facial emotions (angry, happy), actions (fist bump, punch), and varying video clip durations (short, medium, long). Video duration conditions were aggregated on basis of the number of added frames. Short duration relates to 1–5 added frames, medium duration to 6–10 added frames and long duration to 11–15 added frames. Error bars depict the standard error of the mean.
(TIF)
