# Supplementary figures and images for: Angry facial expressions bias towards aversive actions
Source: PLoS One. 2021 Sep 1;16(9):e0256912. doi: 10.1371/journal.pone.0256912 (PMC8409676; doi:10.1371/journal.pone.0256912)

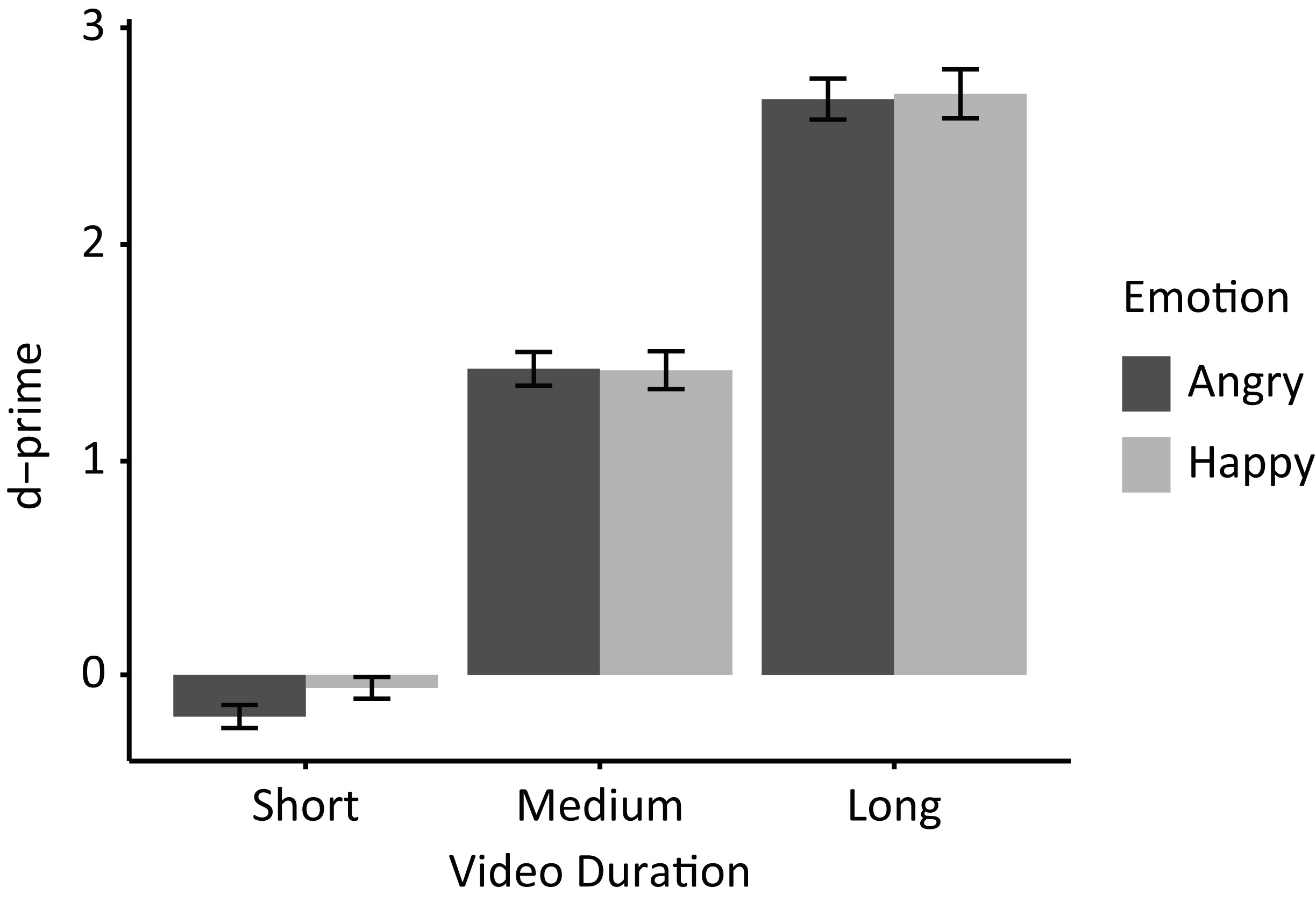

Supplement: S1 Fig — Averaged d-prime values as a function of video durations (short, medium, long) and facial emotions of the agents (angry, happy). Video duration conditions were aggregated on basis of the number of added frames. Short duration relates to 1–5 added frames, medium duration to 6–10 added frames and long duration to 11–15 added frames. Error bars depict the standard error of the mean. (TIF) [file pone.0256912.s001.tif]

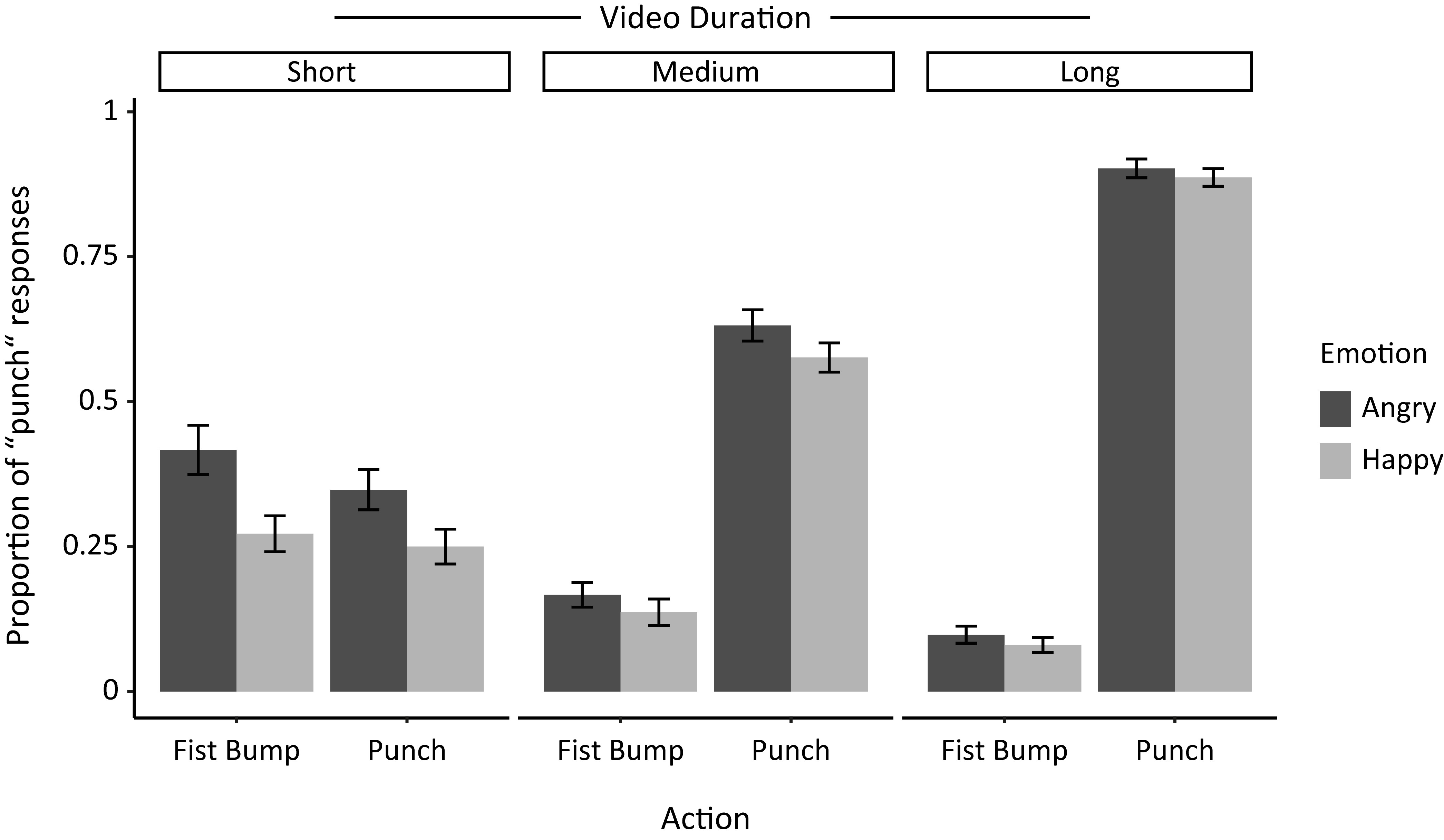

Supplement: S2 Fig — Proportion of punch responses as a function of facial emotions (angry, happy), actions (fist bump, punch), and varying video clip durations (short, medium, long). Video duration conditions were aggregated on basis of the number of added frames. Short duration relates to 1–5 added frames, medium duration to 6–10 added frames and long duration to 11–15 added frames. Error bars depict the standard error of the mean. (TIF) [file pone.0256912.s002.tif]
